# Chinese Herbal Medicine for Reducing Chemotherapy-Associated Side-Effects in Breast Cancer Patients: A Systematic Review and Meta-Analysis

**DOI:** 10.3389/fonc.2020.599073

**Published:** 2020-12-09

**Authors:** Sha Li, Tsz-him So, Guoyi Tang, Hor-Yue Tan, Ning Wang, Bacon Fung Leung Ng, Chris Kam Wa Chan, Edwin Chau-Leung Yu, Yibin Feng

**Affiliations:** ^1^ Li Ka Shing Faculty of Medicine, School of Chinese Medicine, The University of Hong Kong, Hong Kong, Hong Kong; ^2^ Department of Clinical Oncology, Li Ka Shing Faculty of Medicine, The University of Hong Kong, Hong Kong, Hong Kong; ^3^ Chinese Medicine Department, Authority Hospital, Hong Kong, Hong Kong; ^4^ Division of Nephrology, Department of Medicine, Li Ka Shing Faculty of Medicine, The University of Hong Kong, Hong Kong, Hong Kong; ^5^ Hong Kong Association for Integration of Chinese-Western Medicine, Hong Kong, Hong Kong

**Keywords:** herbal medicine, chemotherapy, side effect, breast cancer, meta-analysis

## Abstract

**Background:**

Chemotherapy usually induces a variety of side-effects in cancer treatment as it cannot tell normal cells apart from cancer cells and kills both. Chinese herbal medicine (CHM) has been regarded as a potential effective intervention for relieving the side-effects of chemotherapy in breast cancer patients.

**Objective:**

This study aims to conduct a comprehensive systematic review and meta-analysis to evaluate the efficacy of CHM as adjuvant therapy for reducing the chemotherapy-induced side-effects in the treatment of breast cancer.

**Methods:**

Main electronic databases were searched up to May 2020 for Randomized Controlled Trials (RCTs) evaluating the effect of CHM on breast cancer patients with chemotherapy. The PRISMA statement was adopted in this study and meta-analyses were performed.

**Results:**

The included studies showed unsatisfied quality. Results based on available literature indicated that the adjunctive use of CHM with chemotherapy may reduce the chemotherapeutic agents-associated adverse events, including nausea and vomiting, diarrhea, alopecia, myelosuppression, and impaired immune function.

**Conclusion:**

A confident conclusion could not be have due to the lack of large scale and high quality trials.

## Introduction

Breast cancer is the most common female malignancy, impacting 2.1 million women each year (1). It is the leading cause of cancer-related death in women worldwide, accounting for 23% of all cancers and 14% of cancer-related deaths (2). Chemotherapy plays a crucial role in the treatment of breast cancer (3). It is extensively used in combination with surgery and radiation therapy. Chemotherapy could prolong lifetime and improve survival of cancer patients on one hand, whereas it also causes problematic side-effects, causing a variety of discomfort and burden for the patients (3, 4). The chemical drugs that are selectively destructive to tumor tissues are used in chemotherapy, but these agents cannot tell normal cells apart from cancer cells and kills both, which results in negatively impact compliance with cancer therapy (5). Therefore, developing interventions to alleviate the adverse side-effects induced by chemotherapy and enhance the well-being of patients are urgently needed in clinic.

Notably, the widely use of Chinese Herbal Medicine (CHM) in cancer patients attracts extensive attentions in recent years (6–8). An accumulative number of cancer patients take CHM as complementary and alternative medicine during chemotherapy to reduce side-effects and symptom discomfort as well as to enhance the body’s immune defences (7, 9). As a matter of fact, the use of Traditional Chinese medicine (TCM)-based CHM for breast cancer has been described for more than 2,000 years in China by TCM physicians (10, 11). It is considered that CHM shows the potency to alleviate the toxicities of chemotherapy agents and control the side-effects, such as improves the patient’s quality of life (QoL), prevents recurrence, and prolongs survival (12–15). Therefore, we performed this systematic review and meta-analysis to evaluate the efficacy of CHM as an adjunctive therapy to reduce side-effect of chemotherapy for breast cancer treatment.

## Methods

This systematic review was performed in accordance with the Preferred Reporting Items for Systematic Reviews and Meta-Analysis (PRISMA). Protocol of this study was registered in International Prospective Register of Systematic Reviews (PROSPERO) with registration number CRD42020186977.

### Search Strategy

Main electronic databases including PubMed, ISI Web of Knowledge, EMBASE, CINAHL Plus, AMED, Cochrane Library, China National Knowledge Infrastructure (CNKI), WanFang Data, Chongqing VIP (CQVIP) and SinoMed since the inception of each database up to end of May 2020. The following terms were searched in the databases: (Chinese herbal medicine OR Chinese Medicine OR herbal medicine OR materia medica OR medicinal plants OR herbs OR plant extract OR phytotherapy OR alternative medicine OR complementary medicine) AND (breast cancer or breast tumor or mammary cancer or breast carcinoma) AND (chemotherapy), with slight modifications for individual searches to suit the instructions of different databases.

### Study Selection

#### Types of Studies

The review will include randomized controlled trials (RCTs) using a two-arm or three-arm parallel design for breast cancer patients with chemotherapy-induced side-effects.

#### Exclusion Criterion

Cross-over trials are not appropriate when an intervention can have a lasting effect that compromises entry to subsequent periods of the trial, or when a disease has a rapid evolution. In our study, CHM always showed a lasting effect on patients, and also, the progression of breast cancer could be fast. Thus, cross-over trials will be excluded. Any study with a sample size of no more than 10 patients will be excluded from this review. Moreover, studies with poor methodological quality (Jadad score = 0/1/2) will also be excluded.

#### Participants

Trials including participants that meet the diagnostic criteria of breast cancer will be included regardless of the stage of cancer. All eligible participants will be included in this study regardless of their gender age, gender or race. Trials with participants who are not appropriate to receive Chinese medicine therapy, such as bleeding disorder or allergy and other additional severe diseases will be excluded.

#### Interventions

CHM administered in different dose types, such as decoction, herbal preparation or extract, patented herbal formula, or herbal compounds. CHM may be administrated before or during or after chemotherapy regardless of treatment course *via* oral or injection. In this review, CHM *VS* placebo, CHM alone *VS* conventional western medicine for reducing side-effects of chemotherapy, or CHM combined with conventional western medicine *VS* conventional western medicine alone will be included.

### Outcome Measures

- vomiting and nausea: frequency and severity of vomiting/nausea;
- diarrhea: frequency and severity of diarrhea
- constipation: frequency and severity of constipation
- alopecia
- myelosuppression: reduction of WBC and blood platelet count
- impaired immune function: CD3+, CD4+, CD8+, CD4/CD8 ratio, NK cells population

### Data Extraction and Assessment of Methodological Quality

Methodological quality of pooled studies was evaluated by using the risk of bias tools according to Cochrane Handbook version 5.1.0. There are six items of risk of bias for assessing the methodological quality of RCTs: selection bias (random sequence generation and allocation concealment), performance bias (blinding of participants and personnel) and detection bias (blinding of outcome measurement), attrition bias (incomplete outcome data), reporting bias (selective reporting), and other bias. Each item was ranked as low risk, unclear risk, and high risk. For random sequence generation, a low risk of selection bias will be judged if a random component in the sequence generation process such as using a computer random number or a random number table is described while a high risk of selection bias will be considered if a non-random component in the sequence generation process is provided. For allocation concealment, a low risk of selection bias will be judged if the investigators enrolling participants could not foresee assignment, for example, central allocation sequentially numbered, opaque, sealed envelopes, or sequentially numbered drug containers of identical appearance were used, while a high risk of bias will be considered if investigators enrolling participants could possibly foresee assignments, for example, envelopes were unsealed or non-opaque. In terms of performance bias, a low risk was considered when blinding of participants was performed or we judged that the outcome will not be influenced even there was incomplete blinding. Similarly, there is low risk of detection bias when the blinding of the results’ assessment was conducted or we judged that the results will not be influenced by lack of blinding. A low risk of attrition bias will be judged if there were no missing outcome data, and a low risk of reporting bias will be considered when the study protocol is available, or it is clear that the paper include all expected outcomes if the study protocol is not present. Two authors assessed the methodological quality of all trials independently and any disagreements were solved by the third author consensus.

### Data Analysis

Cochrane Collaboration Review Manage software (RevMan 5.4) was used for data analysis. Dichotomous data were reported as relative risk (RR) with 95% confidence intervals (95% CI), whereas continuous data were expressed as mean ± standard deviation (SD). In our recruited studies, chemotherapy-induced nausea and vomiting, diarrhea, constipation, alopecia, and myelosuppression were assessed by grading the severity and frequency, presenting as dichotomous data, while the evaluation of the immune function was performed by detecting the population of T cells such as CD3+/CD4+/CD8+ subtypes and Natural Killer (NK) cells, which are presented as mean ± SD. *I* ^2^ was used to measure the heterogeneity; when the heterogeneity was significant in the included studies (*I* ^2^ > 50%), a random-effect model would be considered; otherwise the fix-effect model was adopted. If *I* ^2^ < 0.05, the differences between the CHM treatment groups and control groups were regarded as statistically significant. In the present study, we performed sub-group analysis based on the principles of TCM treatment of breast cancer, which mainly include three aspects: strengthening the body (Fu Zheng, +), eliminating pathogenic factors (Qu Xie, −), or both (Fu Zheng and Qu Xie, ±).

## Results

### Characteristics of the Included Studies

**Figure 1:**
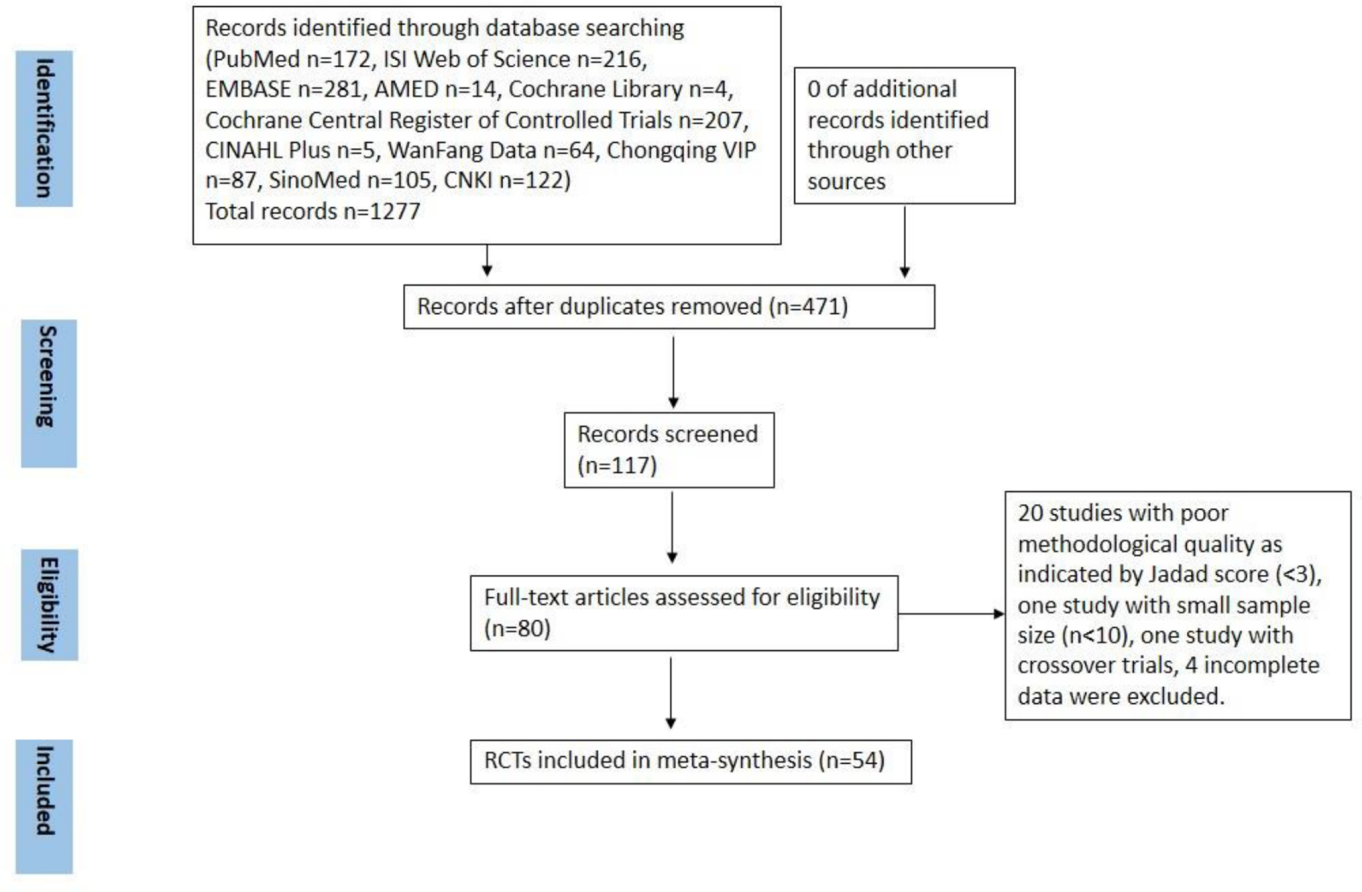
Study flow diagram.

In initial database searching, total 1,288 studies were retrieved. After duplicated studies removal, there remained 471 records. After further review of the abstracts, 354 records were excluded with reasons of animal studies, retrospective studies, literature reviews or not relevant clinical studies with CHM and chemotherapy. Then only 117 records are eligible for further consideration, and only 80 full-text are available. After full-text review, 20 studies with poor methodological quality as indicated by Jadad score (<3), one study with small sample size (n < 10), one study with crossover trials, four with incomplete data were excluded. So there were a total of 54 studies included for meta-analysis in our study. Among them, three studies were retrieved from English the database and 51 from Chinese database. The flow diagram of the study selection was shown in **Figure 1**.

**Figure 2:**
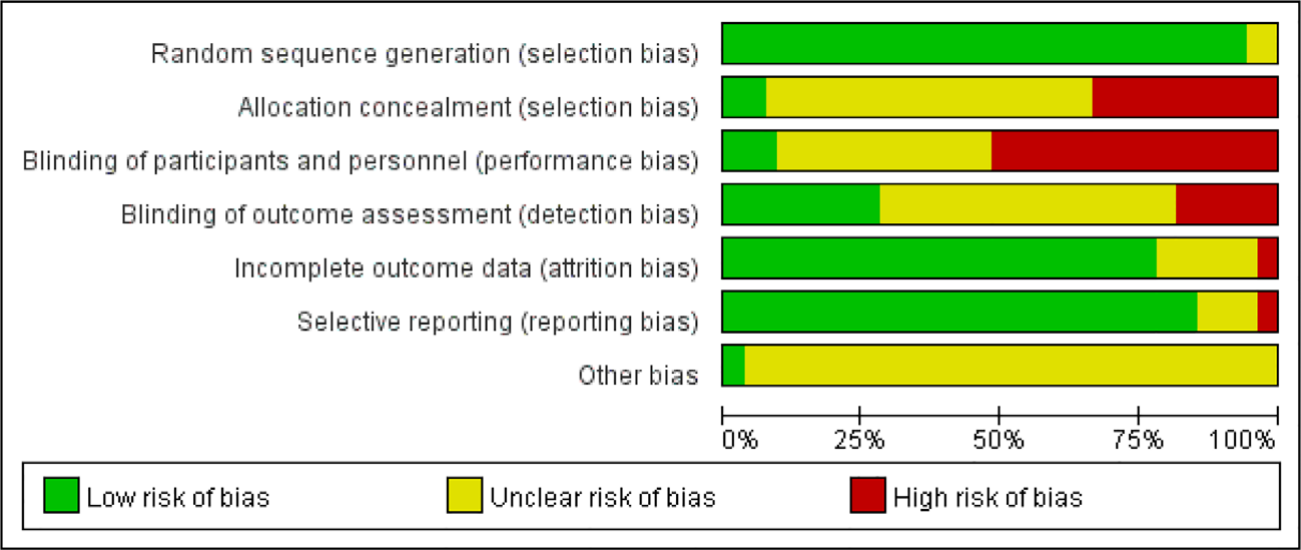
Risk of bias graph.

**Figure 3:**
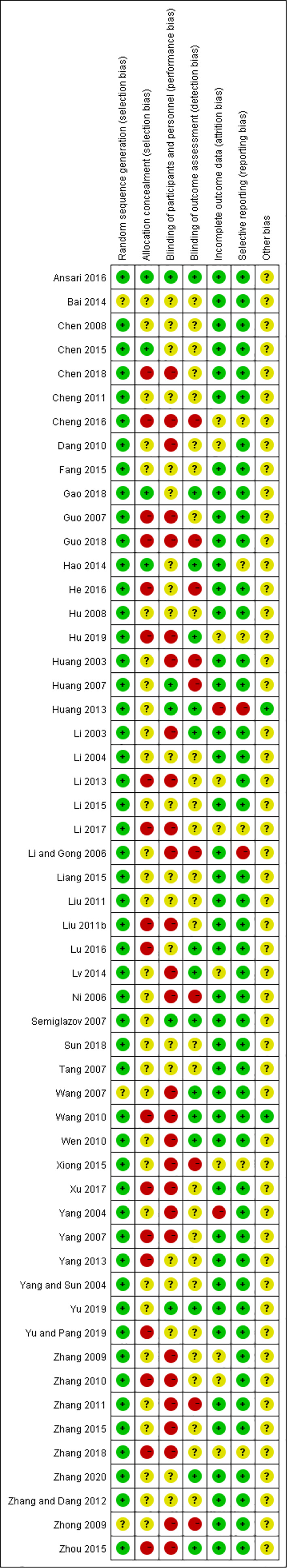
Risk of bias summary.

A total of 4,032 patients were enrolled in these included studies, among which 2,081 patients received CHM interventions while 2,002 patients participated in the control group, and 49 patients dropped out. All included patients in these studies were diagnosed *via* pathological examination. The baseline characteristics of the included studies were listed in **Table 1**. All those studies showed a comparable baseline data. The risk of bias of recruited studies was evaluated by the tool of Cochrane Collaboration. Most of pooled studies performed a random grouping referring to a random number table or using a computer random number, indicating the low risk of selection bias in terms of random sequence generation (**Figures 2** and **3**). In most of these studies, the allocation concealment and blinding of participants and personnel as well as blinding of outcome assessment were not mentioned, resulting in the unclear/high risk of selection bias, performance bias and detection bias, as shown in **Figures 2** and **3**.

**Table 1:** The characteristics of included studies (CHM belongs to Fu Zheng (+), Qu Xie (−), or Fu Zheng and Qu Xie (±)).

Study	Sample size (T + C)/drop-outs	Diseasestage	Chemotherapy	Experimental CHMs	Control group intervention	Duration	Outcome measures	Jadad scale
Ansari et al. (16)	150(57 + 62)/31	Not mentioned	AC, CAF and TAC	Ginger powder, (−)	Placebo capsules	Twice a day for 3 days	Frequency and grade of CINV	5
Bai et al. (17)	64(32 + 32)/0	I–IV	TEC	Jinlong capsule, (−)	Not mentioned	12 weeks	Tumor response and chemotoxicity, KPS	3
Chen et al. (18)	60(30 + 30)/0	I–III	CEF : CTX/EPI/5-Fu	Danggui Buxie decoction, (+)	No placebo	24 weeks	QoL, chemotoxicity, T cells population	3
Chen et al. (19)	60(28 + 29)/3	I–III	GP : GCB + PDD	Sugan Jianpi Sanjie compound, (±)	Not mentioned	6 weeks	Chemotoxicity, KPS, T cells population	3
Chen et al. (20)	56(28 + 28)/0	Not mentioned	Chemotherapy without mention of specific drugs	Yiqi Jianpi Hewei Therapy. (+)	Ondansetro, and Dexamethasone Sodium Phosphate	10 days	Gastrointestinal adverse reaction (CTCAE3.0V), QOL, recurrence rate	3
Cheng (21)	50(26 + 24)/0	Not mentioned	TP : TAX/PDD	Fuzheng Kangai compound, (±)	No placebo	8 weeks	Tumor response and chemotoxicity, KPS	3
Cheng and Zhang (22)	40(20 + 20)/0	IV	XD	Fuzheng Jiedu formula, (±)	Not mentioned	6 weeks	KPS	3
Dang and Wang (23)	48(28 + 20)/0	I–III	CTF	Aidi injection. (+)	Not mentioned	9 weeks	Tumor response, KPS, cardiotoxicity, chemotoxicity	3
Fang and Jia (24)	30(16 + 14)/0	II–IV	CE : CTX + EPI	Herbal formula, (±)	No placebo	12 weeks	Tumor response and chemotoxicity, KPS	3
Gao et al. (25)	132(66 + 66)/0	Not mentioned	AF/AC	Fuzheng Jiandu Xiaoliu Decoction, (±)	No treatment	6 months	T cells population, gastrointestinal reaction	3
Guo et al. (26)	30(10 + 20)/0	IV	CEF	Modified Sijunzi Decoction. (+)	No treatment	3 weeks	T cells population	3
Guo (27)	86(43 + 43)/0	I–III	CEF	Yiqi Yangxie Shengjin formula, (±)	Not mentioned	9 weeks	Myelosuppression, WHO standard	3
Hao et al. (28)	96(49 + 47)/0	I–III	CEF	Guilu Erxian Decoction, (±)	Not mentioned	18 weeks	T cells population, KPS	3
He and Ruan (29)	80(40 + 40)/0	II–III	AC, CAF, EC	Xiangsha Liujunzi and Jupi Zhuru decoction. (+)	Not mentioned	5 days	European society of clinical oncology recommended standards	3
Hu and He (30)	68(34 + 34)/0	I–IV	TA/CAF	Yiqi Jianpi Shugan Decoction, (±)	Placebo	12 weeks	Changes of cell immune function and quality of life (Karnofsky)	3
Hu et al. (31)	52(28 + 24)/0	II–IV	CAF : CTX/ADM/5-Fu	Yiqi Huoxie decoction. (+)	No placebo	20 days	Tumor response and chemotoxicity	3
Huang et al. (32)	66(37 + 29)/0	II–IV	CMF : CTX/MTX/5-Fu	Modified Bazhen decoction. (+)	No placebo	6 weeks	Tumor response, KPS and chemotoxicity	3
Huang et al. (33)	60(30 + 30)/0	IV	CTF	Jianpi Xiaoji decoction, (±)	Not mentioned	More than 4 weeks	quality of life (Karnofsky), T cells population	4
Huang et al. (34)	60(30 + 30)/0	IV	CEF	Huangqi injection. (+)		18–24 weeks	Tumor response, KPS, chemotoxicity	3
Li and Han (35)	85(44 + 41)/0	I–IV	CEF	Fuzheng Xiaoliu compound, (±)	No treatment	9 weeks	Tumor response and chemotoxicity, tumor marker	3
Li and Gong (36)	52(32 + 20)/0	I–III	CEF	Aidi injection. (+)	Not mentioned	9 weeks	Tumor response, chemotoxicity	3
Li and Zhong (37)	120 (60 + 60)/0	I–IV	FAC	Bushen Shugan Huatan decoction, (±)	Vitamin et al	18 weeks	WHO standard: nausea and vomiting, myelosuppression, survival rate	3
Li et al. (38)	101(61 + 40)/0	I–III	CMF	Rukang I prescription. (+)	Not mentioned	6–9 weeks	Tumor response, KPS, chemotoxicity	3
Li et al. (39)	75(40 + 35)/0	I–IV	NE : NDP/EPI	Shenqifuzheng injection. (+)	Not mentioned	12 weeks	Tumor response and chemotoxicity	3
Li and Li(40)	128(64 + 64)/0	I–II	postoperative chemotherapy of ROBC	Fuzheng Xiaoliu decoction, (±)	Placebo	18 weeks	T cells, inflammation marker, QoL	3
Liang et al. (41)	98(48 + 50)/0	I–IV	TAC	Huaier Granule, (−)	Not mentioned	24 weeks	T cells population and survival rate	3
Liu et al. (42)	66(31 + 35)/0	I–II	CAF	Tianzhicao capsule, (±)	Not mentioned	24 weeks	KPS, chemotoxicity	3
Liu et al. (43)	50(25 + 25)/0	I–IV	TE : TAX/EPI	Renshen Yangrong decoction. (+)	No placebo	6 weeks	KPS, T cells population	3
Lu et al. (44)	90(45 + 45)/0	I–IV	FEC	Huaier granule, (−)	No treatment	3 weeks	T cells and NK cells	4
Lv et al. (45)	54(27 + 27)/0	IV	FAC	Yiqi Huoxie Huayu decoction, (±)		12–16 weeks	Tumor response, KPS, chemotoxicity and immune function	3
Ni (46)	57(30 + 27)/0	IV	Docetaxel + THP	Gaolisheng injection. (+)	Not mentioned	13 weeks	Tumor response and chemotoxicity	3
Semiglazov et al. (47)	352(169 + 168)/15	pT1–T3 pN0-N+ (0–10 positive lymph nodes) pM0	CMF	0.5 ml of the study medication PS76A2 (aqueous mistletoe extract). (+)	0.5 ml placebo	Twice weekly for 16 to 24 consecutive weeks	FACT-G scale	3
Sun et al. (48)	74(37 + 37)/0	Not mentioned	CEF or TEC	Shugan Jianpi, Fuzheng Xiaoyu decoction, (±)	granisetron hydrochloride	15 days	QLQ - C30	3
Tang et al. (49)	60(30 + 30)/0	I–IV	CEF : CTX/EPI/5-Fu	Yiqi Jiedu decoction, (±)	No placebo	12 weeks	T cells population and KPS	3
Wang (50)	60(30 + 30)/0	II–III	CEF	Taohong Siwu decoction, (±)	Not mentioned	9 weeks	QoL and chemotoxicity	3
Wang (51)	40(20 + 20)/0	II–IV	TE	Yiqijianpi Huayujiedu decoction, (±)	Not mentioned	8 weeks	Tumor response, QoL and chemotoxicity	4
Wen et al. (52)	60(30 + 30)/0	IV	TA	Fuzheng Xiaoyan prescription, (±)	Not mentioned	3 weeks	Tumor response, QoL	4
Xiong et al. (53)	92(50 + 42)/0	I–III	TAC	Huaier granule, (−)	Placebo	18 weeks	T cells population and NK cells	3
Xu (54)	60(30 + 30)/0	Not mentioned	Doxorubicin, Cyclophosphamide, 5-Fu	Sini Decoction. (+)	Not mentioned	2 weeks	Heart toxicity, WHO standard	3
Yang (55)	59(31 + 28)/0	IV	NVB + THP	Aidi injection. (+)	Not mentioned	6 weeks	Tumor response, KPS and immune function	3
Yang and Sun (56)	51(27 + 24)/0	Not mentioned	CMF : CTX/MTX/5-Fu	Modified Liujunzi decoction. (+)	No placebo	9 weeks	Tumor response and chemotoxicity	3
Yang et al. (57)	38(20 + 18)/0	I–IV	CEF : CTX/EPI/5-Fu	Taohongsiwu decoction, (±)	No placebo	9 weeks	Tumor response and chemotoxicity	3
Yang (58)	52(26 + 26)/0	Not mentioned	CEF or TEC	Yiqi Jianpi Hewei Therapy. (+)	Ondansetro, and Dexamethasone Sodium Phosphate	10 days	Gastrointestinal adverse reaction (CTCAE3.0V)	3
Yu and Pang (59)	164(82 + 82)/0	III–IV	CAF	Fuzheng Xiaozheng Qudu formula therapy, (±)	Not mentioned	24 weeks	KPS	3
Yu et al. (60)	93(47 + 46)/0	I–IV	Docetaxel and adriamycin	Xiaoaiping tablet, (−)	Placebo	2 weeks during each chemotherapy cycle	QoL; BWB, EWB, FWB, PWB, and SWB scores	5
Zhang (61)	50(26 + 24)/0	I–IV	NP : NDP/PDD	Herbal formula, (±)	No placebo	4 weeks	Tumor response and chemotoxicity, KPS	3
Zhang (62)	70(40 + 30)/0	Not mentioned	CTX+EPI	Fuzheng Jiandu Xiaoliusan formula, (±)	Not mentioned	5 months	Adverse effect, CD4, CD8	3
Zhang and Dang (63)	60(30 + 30)/0	I–IV	NP: NDP+PDD	Fuzheng Guben compound. (+)	Not mentioned	4 weeks	RECIST, KPS, chemotoxicity	3
Zhang and Wang (64)	110(55 + 55)/0	I–III	FEC	Shenmai injection. (+)	No treatment	19 weeks	TCM symptom score and T cells population	4
Zhang et al. (65)	80(40 + 40)/0	I–III	CEF	Huangqi Taohong decoction, (±)	Not mentioned	18 weeks	Immune system	3
Zhang et al. (66)	45(23 + 22)/0	III–IV	CTF	Fuzheng Quyu Jiedu prescription, (±)		6 weeks	QoL and immune system	3
Zhang (67)	50(25 + 25)/0	Not mentioned	GT	Shugan Jianpi Yishen decoction, (±)	Not mentioned	15 weeks	Tumor response (RECIST), Chemotoxicity (CTCAE v3.0)	3
Zhong (68)	40(20 + 20)/0	I–IV	TE	Shugan Tiaoli Chongren decoction, (±)	Not mentioned	6 weeks	QoL, KPS, and chemotoxicity	3
Zhou (69)	108(54 + 54)/0	II–IV	Not shown	Guipi & Guilu Erxian Decoction, (±)	Placebo	2 months	WBC count	3

AC, Adriamycin + Cyclophosphamide; CAF, Cytoxan + Adriamycin + Fluorouracil; TAC, Taxotere + Adriamycin + Cyclophosphamide; TEC, Taxotere + Epirubicin + Cyclophosphamide; CEF, CTX/EPI/5-Fu, Cyclophosphamide + Epirubicin + 5-Fluorouracil; GP, GCB + PDD: Gemcitabine + Cisplatin; TP, TAX/PDD: Taxotere + Cisplatin; XD, Docetaxel + Capecitabine; CTF, Cyclophosphamide + Pirarubicin + 5-Fluorouracil; CE, CTX + EPI: Cyclophosphamide + Epirubicin; EC, Epirubicin + Cyclophosphamide; TA, Taxotere + Adriamycin; CAF, CTX/ADM/5-Fu: Cytoxan + Adriamycin + Fluorouracil; CMF, CTX/MTX/5-Fu: Cytoxan + Methotrexate + Fluorouracil; FAC, Fluorouracil + Adriamycin + Cytoxan; NE, NDP/EPI: Nedaplatin + Epirubicin; TE, TAX/EPI: Taxotere + Epirubicin; FEC, 5-Fluorouracil (5FU) + Epirubicin + Cyclophosphamide; THP, Taxotere + Herceptin + Perjeta; CMF, Cyclophosphamide + Methotrexate + 5-Fluorouracil; NVB + THP, Vinorelbine + Taxotere + Herceptin + Perjeta; NP, NDP/PDD: Nedaplatin + cisplatin; CTX + EPI, Cyclophosphamide + Epirubicin; GT, Gemcitabine + Paclitaxel.

### Chemotherapy-Induced Nausea and Vomiting

**Figure 4:**
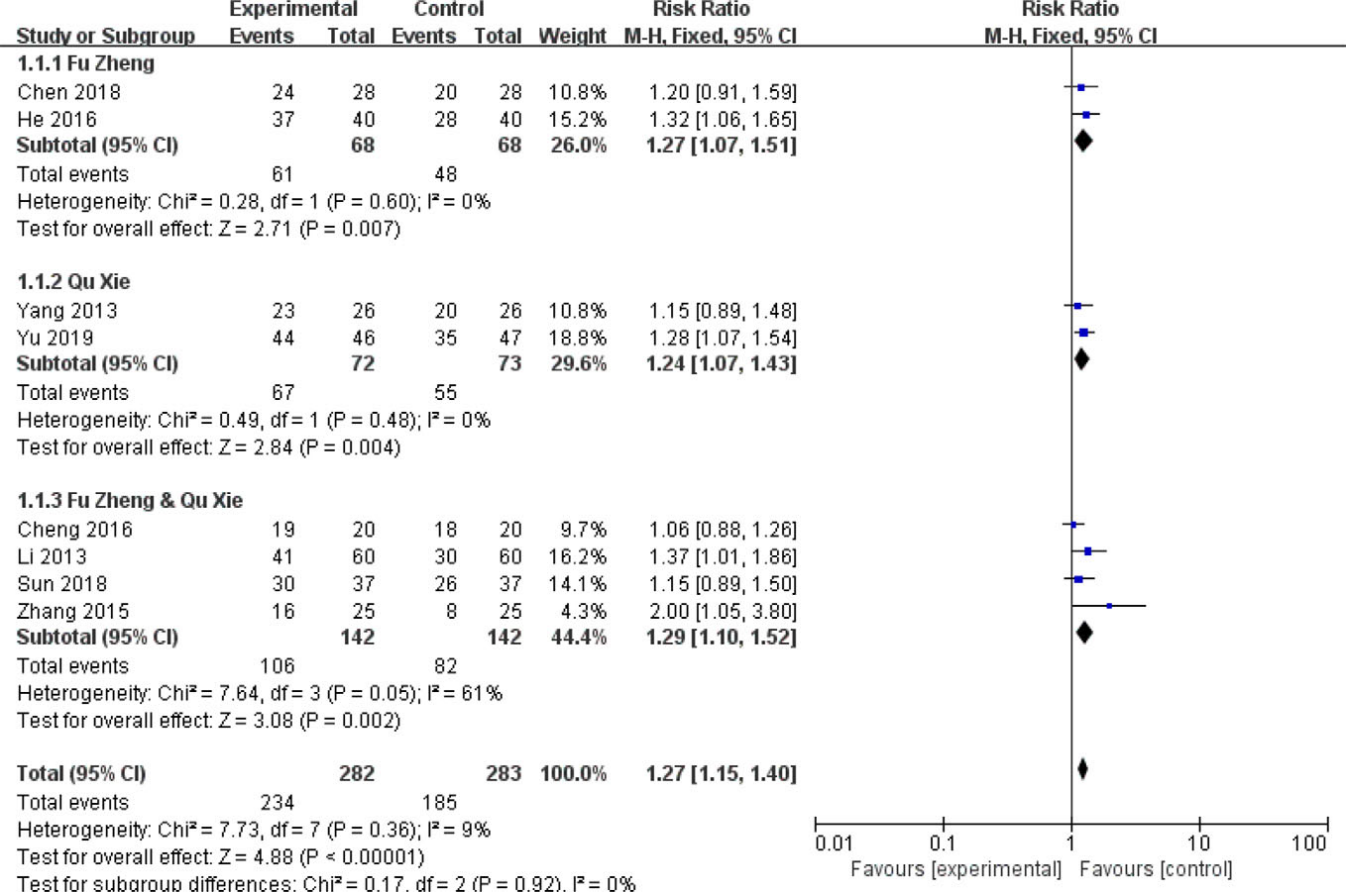
Forest plot of CINV in patients of breast cancer (toxicity grades 0–II).

**Figure 5:**
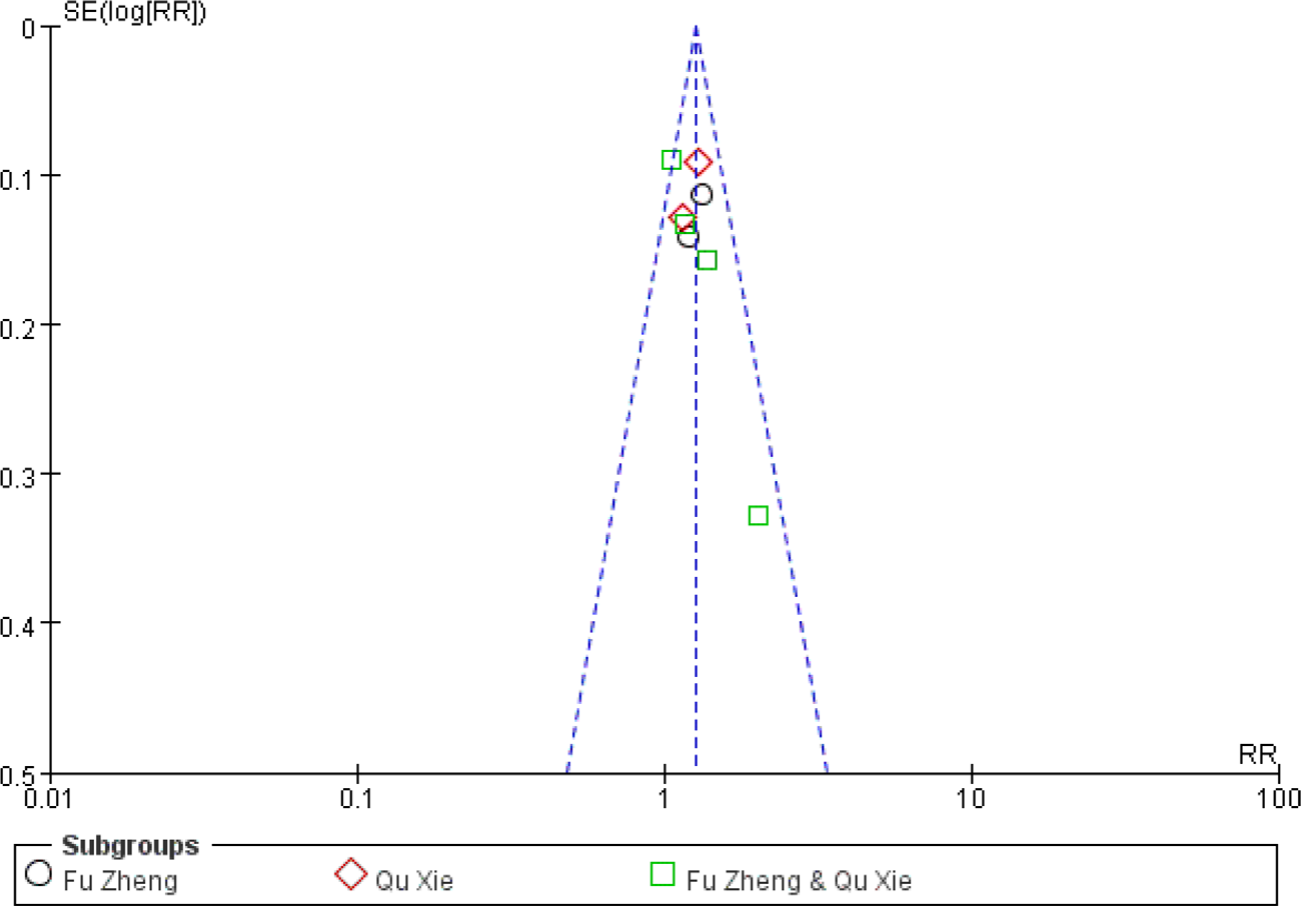
Funnel plot of CINV in patients of breast cancer (toxicity grades 0–II).

**Figure 6:**
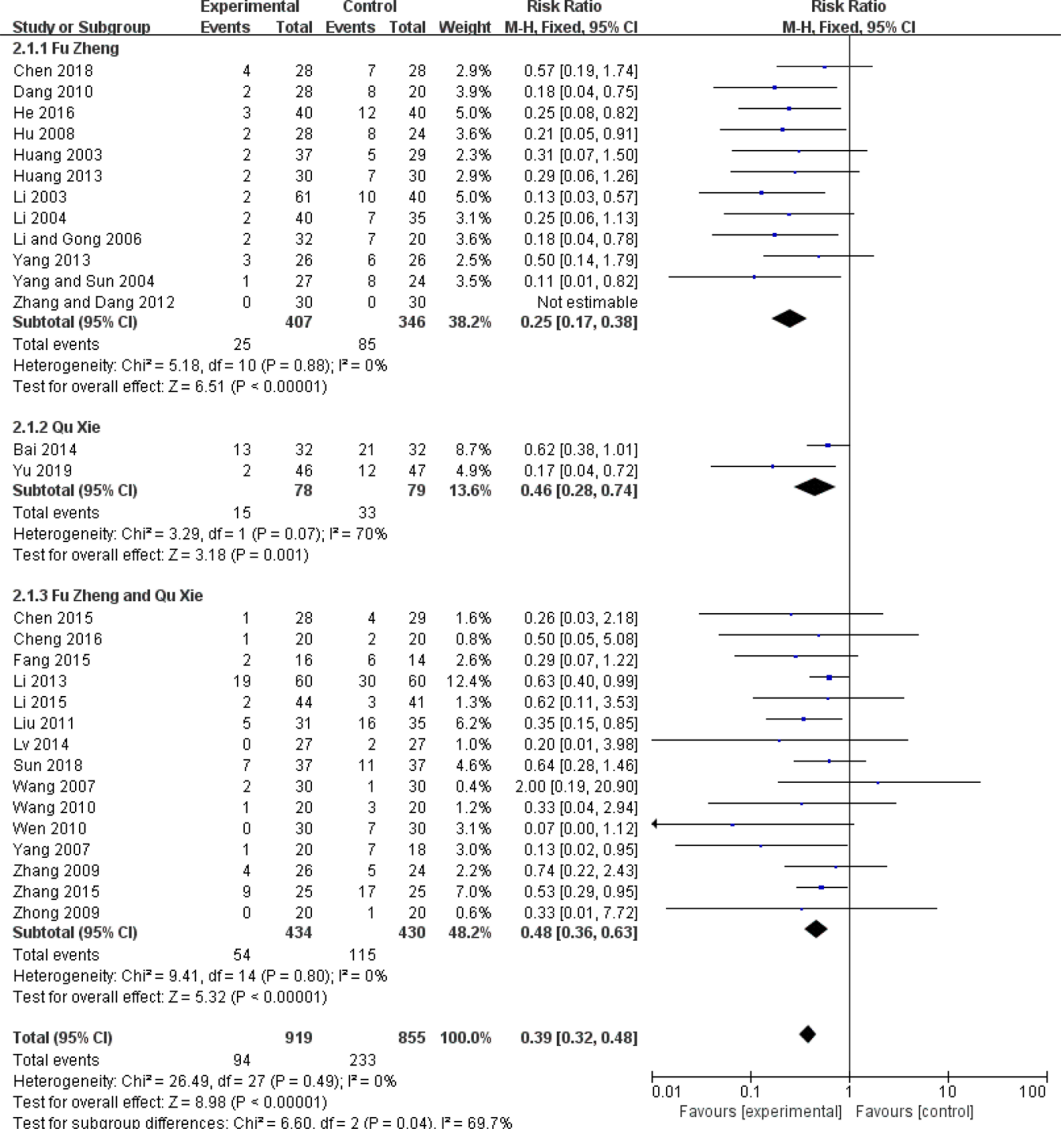
Forest plot of CINV in patients of breast cancer (toxicity grades III–IV).

**Figure 7:**
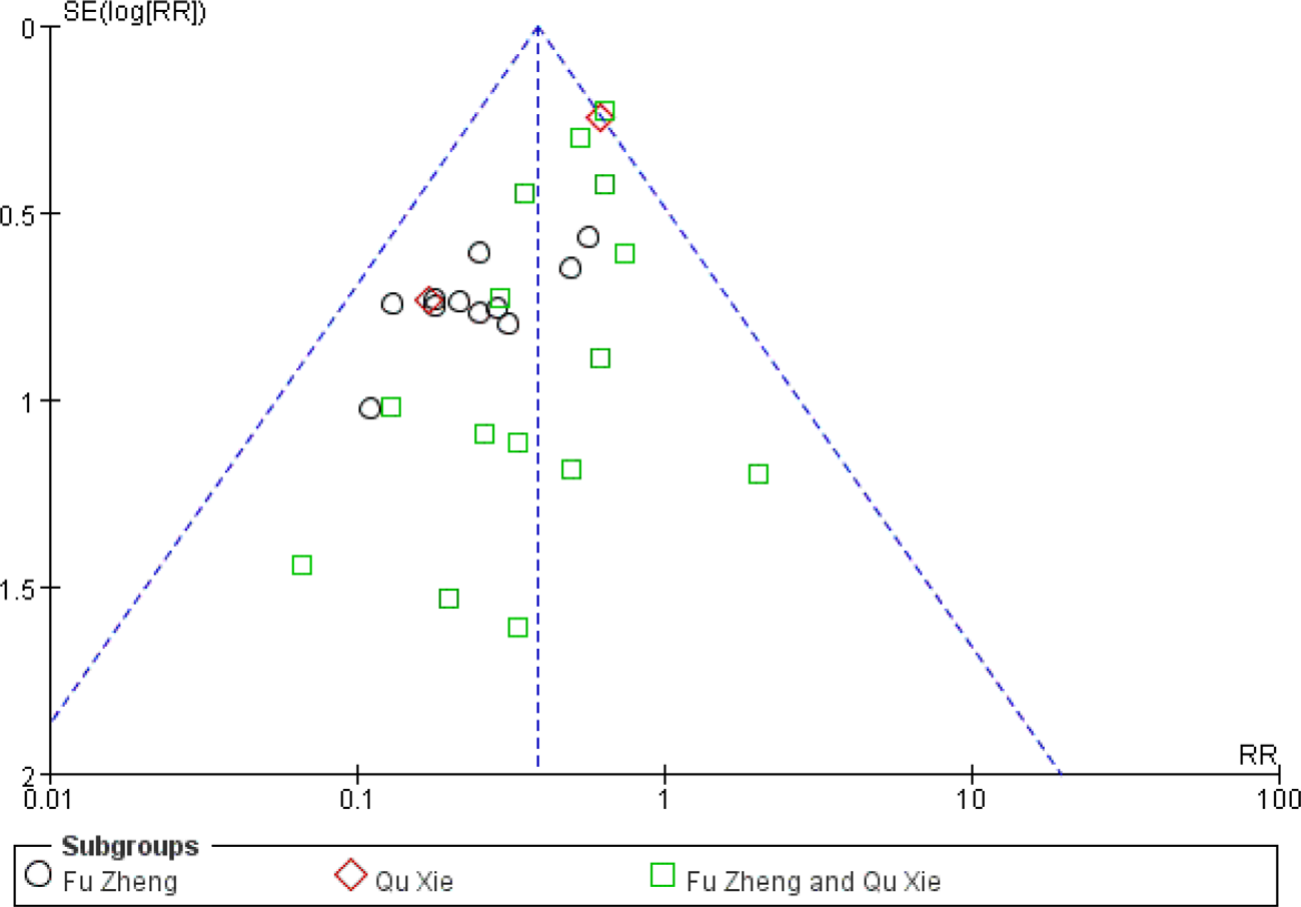
Funnel plot of CINV in patients of breast cancer (toxicity grades III–IV).

Among the side-effects induced by chemotherapy, chemotherapy-induced nausea and vomiting (CINV) is the most common symptom (70, 71). According to the chemotherapy-induced toxicity grading criteria, for example CTCAE version 4, none/one to two episodes/three to five episodes in 24 h was graded as Grade 0/1/2, which could be considered as mild to moderate toxicity, while others such as ≥6 episodes in 24 h are severe toxicity. In certain grade of our pooled studies, there are only zero or one or two events in either the experimental or control groups. To avoid the imprecision of the pooled results due to few events of some grades, combination of these data from several grades is adopted. The frequency of CINV with grades 0–II was significantly higher in the CHM group than in the control group (RR = 1.27, 95% CI = 1.15–1.4, I^2^ = 9%, eight studies (18, 22, 29, 37, 48, 58, 60, 67), 665 patients **Figures 4** and **5**). For each sub-group analysis, the frequency of CINV with grades 0–II was significantly higher in the CHM group than in the control group. Moreover, there is no statistical significance between these sub-groups (p = 0.92). Regarding the frequency of grades III–IV, which indicated severe nausea and vomiting (RR = 0.39, 95% CI = 0.32–0.48, I^2^ = 0%, 28 studies (17, 19, 20, 22–24, 29, 31, 32, 34–39, 42, 45, 48, 50–52, 56–58, 60, 61, 63, 67, 68), 1,774 patients, **Figures 6** and **7**), was lower significantly in patients receiving CHM treatment, suggesting the beneficial effects of CHM in alleviating CINV. Our sub-group analysis showed that the frequency of grades III–IV was significantly lower in the CHM group than in the control group. Notably, there is statistical significance between these sub-groups (p = 0.04), suggesting CHM belonging to different TCM theory showed different magnitude of therapeutic effects. In the study of Ansari et al., the criteria used are the one defined by the authors (grades 0–III); thus we did not include this study to perform the meta-analysis. This study found that ginger reduced vomiting severity from 1.4 ( ± 1.04) to 0.71 ( ± 0.86) in all sessions, but the results showed no statistically significance. Only in patients of the AC sub-group, ginger treatment significantly decreased the severity of vomiting (0.64 ± 0.87) compared to those from the placebo group (1.13 ± 1.12).

### Diarrhea

**Figure 8:**
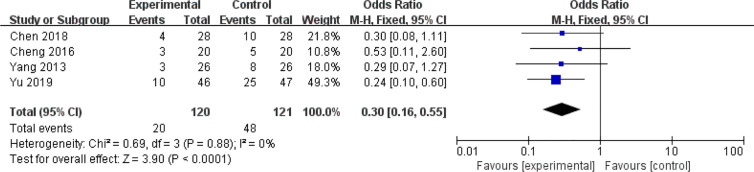
Forest plot of diarrhea in patients of breast cancer (toxicity grades III–IV).

**Figure 9:**
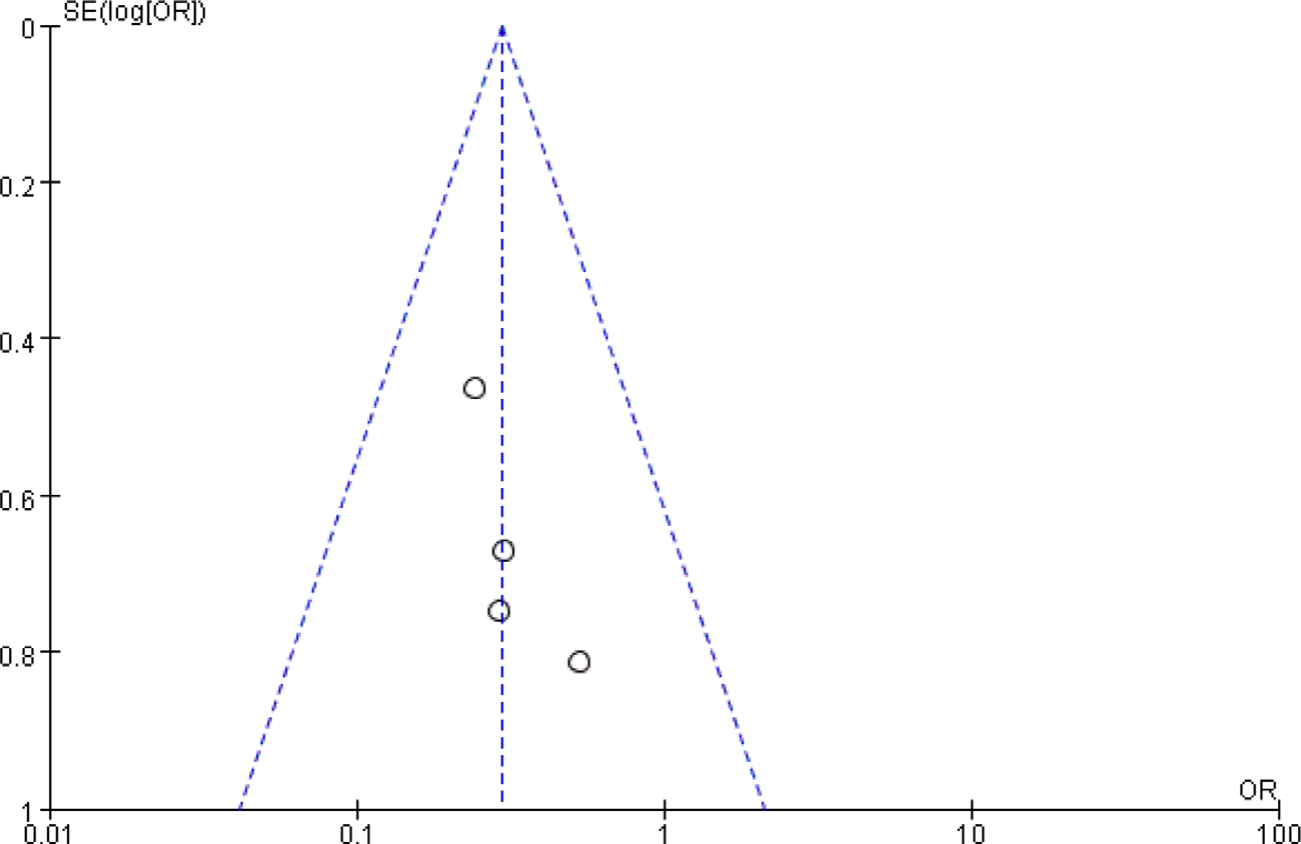
Funnel plot of diarrhea in patients of breast cancer (toxicity grades III–IV).

Another common gastrointestinal adverse effects of chemotherapy is diarrhea, which occurs in up to 60% of cancer patients treated with chemotherapy, and 10% have severe diarrhea (72, 73). As shown in **Figures 8** and **9**, the events of severe diarrhea in the CHM treated group were significantly lowered than that of the control group (RR = 0.3, 95% CI = 0.16–0.55, I^2^ = 0%, four studies (20, 22, 58, 60), 241 patients, **Figures 8** and **9**). Among those four studies, two studies (22, 60) defined the severity of diarrhea by using the World Health Organization (WHO) criteria, while the other two evaluated it according to CTCAE3.0V. Although the criteria used is not the same, they are comparable because both of them defined the severity into five level grades (the higher number, the more severe it is). Of note, the study by Yu et al. (60) showed high quality, which was assessed as low risk of selection bias, performance bias, detection bias, attrition bias, and reporting bias. In this study, Xiaoaiping, a drug composed of the Chinese herb *Marsdeniae tenacissimae* (Z20063919), showed remarkable effects in the reduction of diarrhea.

### Constipation

**Figure 10:**
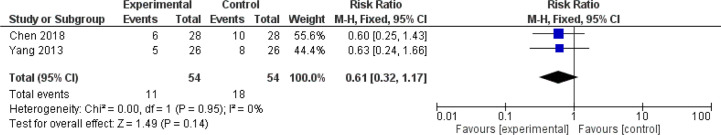
Forest plot of constipation in patients of breast cancer (toxicity grades III–IV).

**Figure 11:**
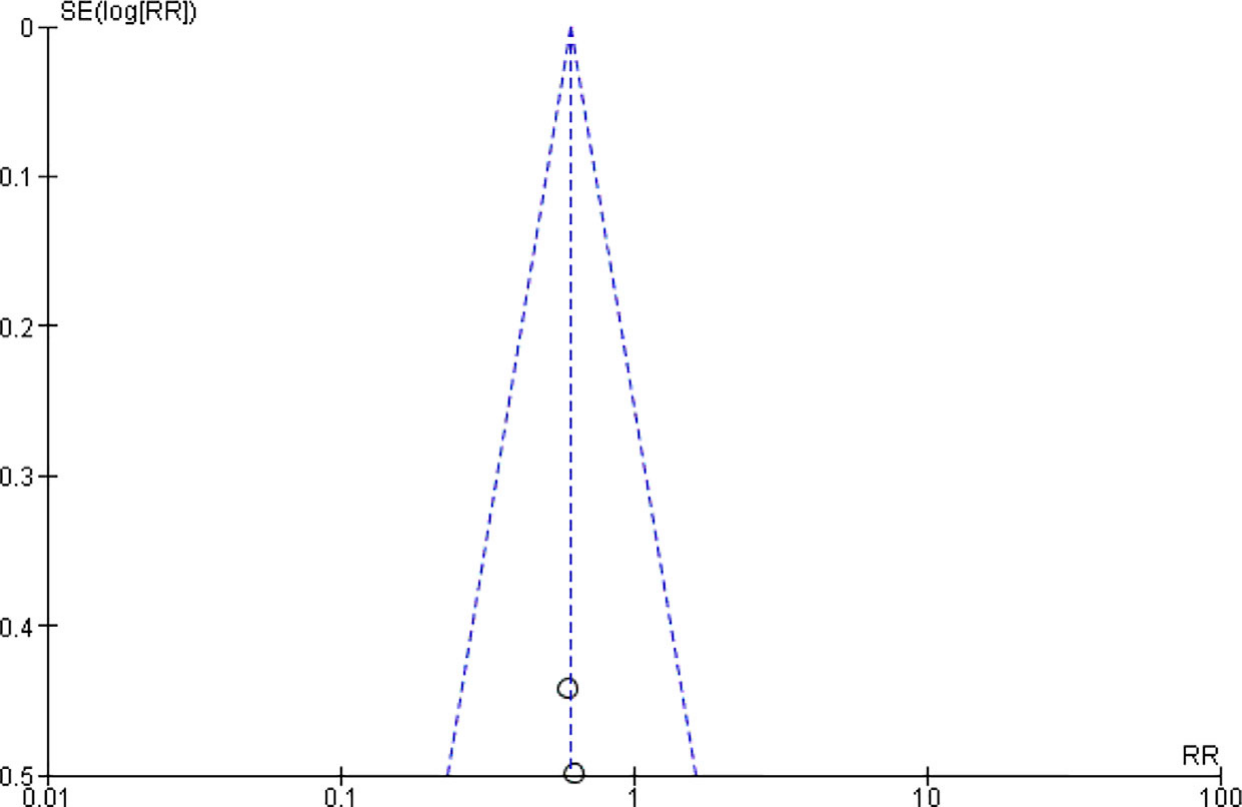
Funnel plot of constipation in patients of breast cancer (toxicity grades III–V).

In some cases, chemotherapy induced the disordered lining of the intestine, resulting in constipation (73). The altered eating habits or reduced activity level due to fatigue and decreased appetite may also cause bowel irregularity and constipation (74, 75). Two of our recruited studies (20, 58) have assessed the efficacy of a traditional CHM named Yiqi Jianpi Hewei on chemotherapy induced constipation in breast cancer patients. **Figures 10** and **11** showed Yiqi Jianpi Hewei therapy alleviated the occurrence rate of severe constipation, while without statistical significant difference (grades II–IV, RR = 0.61, 95% CI = 0.32–1.17, I^2^ = 0%, two studies (20, 58), 108 patients).

### Alopecia

**Figure 12:**
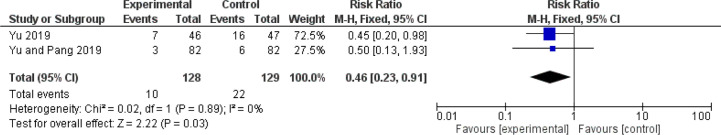
Forest plot of alopecia in patients of breast cancer (toxicity grades III–IV).

**Figure 13:**
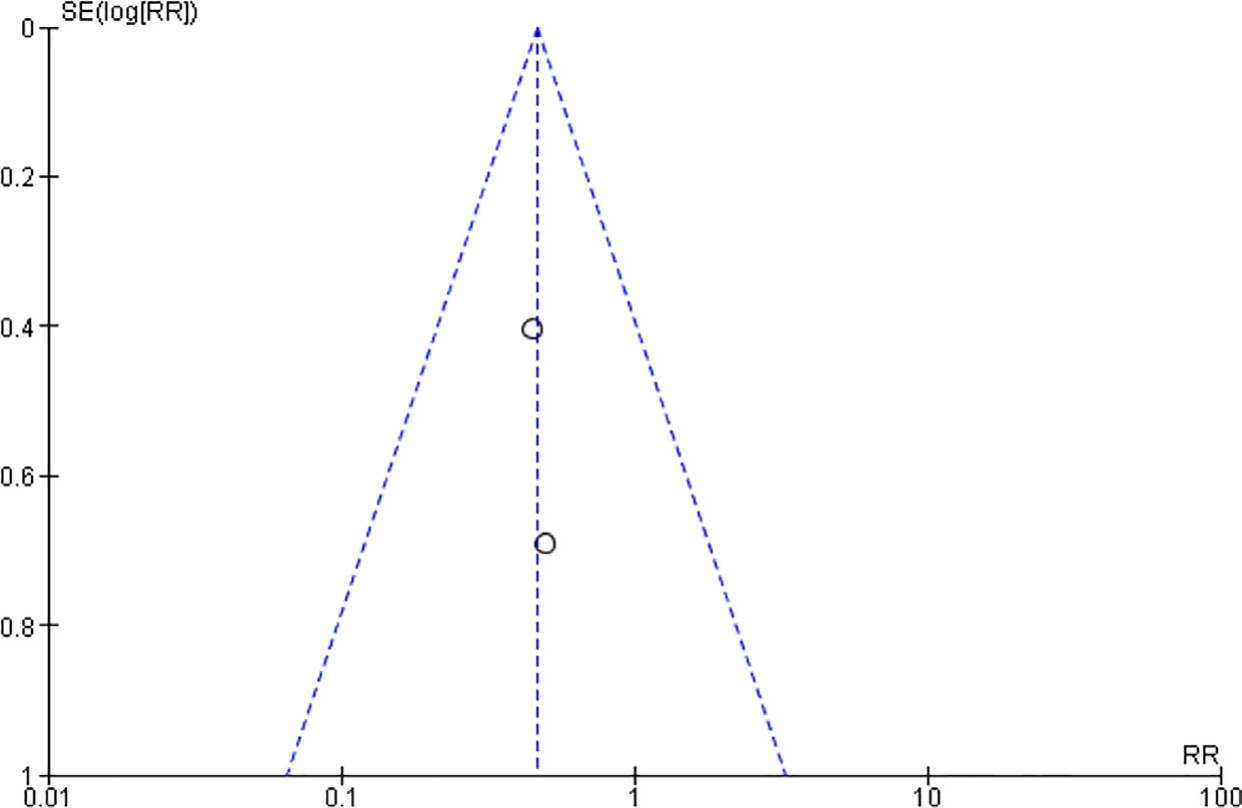
Funnel plot of alopecia in patients of breast cancer (toxicity grades III–IV).

As chemotherapeutic agents generally interfere with cells of rapidly dividing, not only tumor cells but also hair follicles, it may cause alopecia (76, 77). After the chemotherapy is finished, hair commonly grows back but in some cases hair cannot regrow (73, 74). In our recruited studies, two studies (59, 60) enrolled 257 patients evaluated the effect of two CHM on alleviating alopecia. As shown in **Figures 12** and **13**, Xiaoaiping and another herbal formula Fuzheng Xiaozheng Qudu could significantly reduce the events of alopecia caused by chemotherapy in patients with breast cancer (RR = 0.39, 95% CI = 0.17–0.88, I^2^ = 0%). There is another randomized, double-blind, multi-center clinical trial to study the effect and mechanism of YH0618 granule on chemotherapy-induced hair loss in breast cancer patients (78). Because this clinical trial is still on-going, and no completed data is available, this study has to be excluded in this meta-analysis.

### Reduced White Blood Cell and Blood Platelet Count

**Figure 14:**
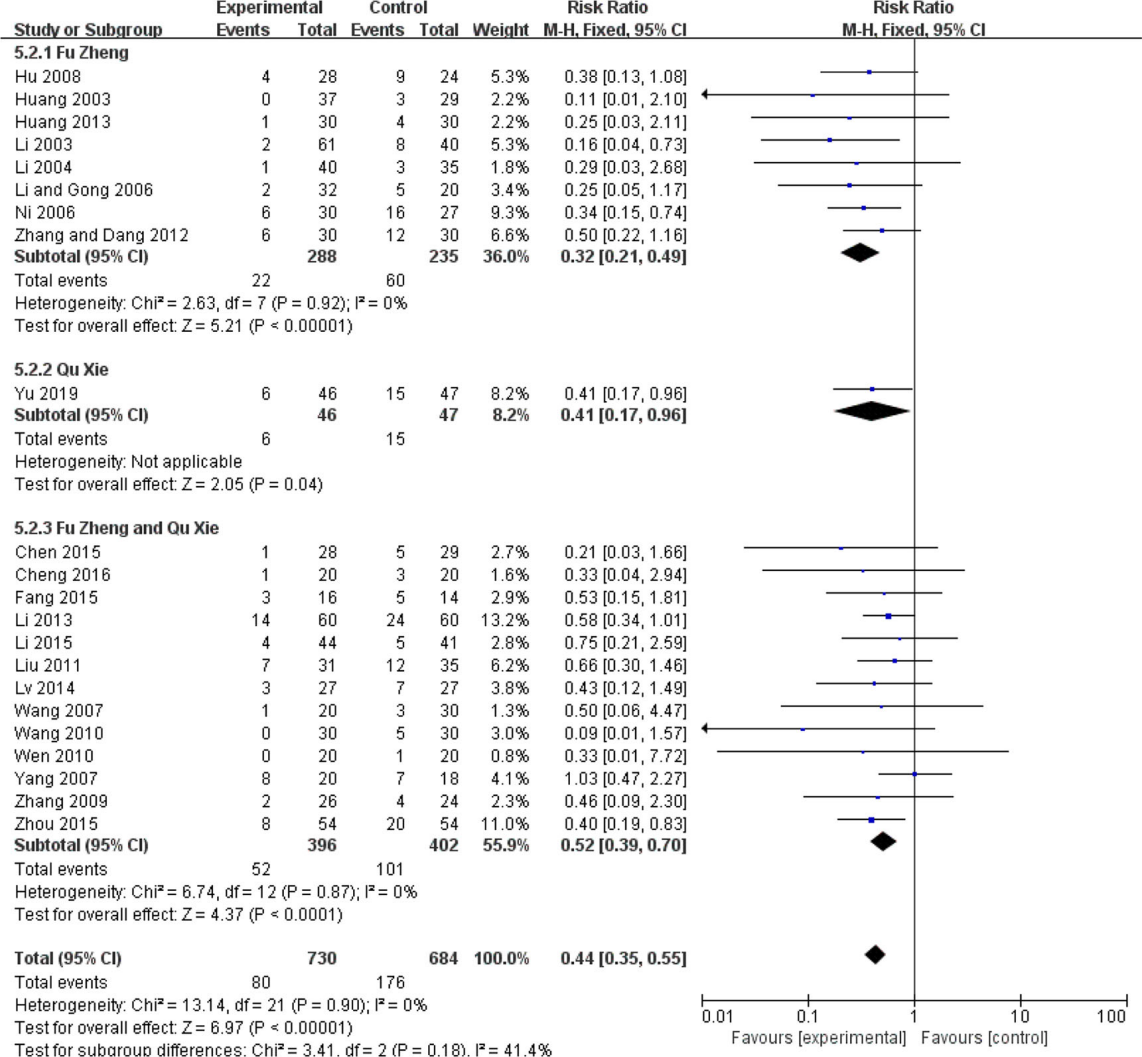
Forest plot of WBC reduction in patients of breast cancer (toxicity grades III–IV).

**Figure 15:**
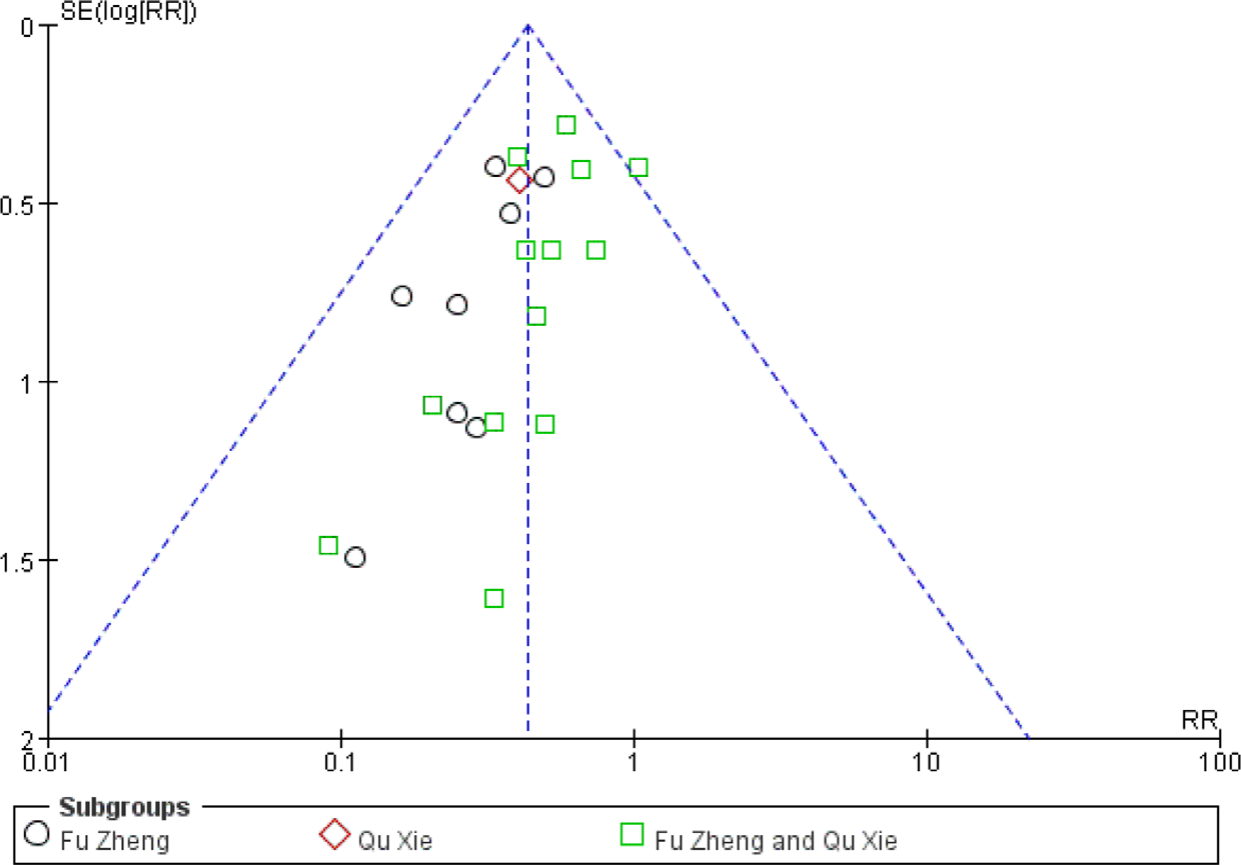
Funnel plot of WBC reduction in patients of breast cancer (toxicity grades III–IV).

**Figure 16:**
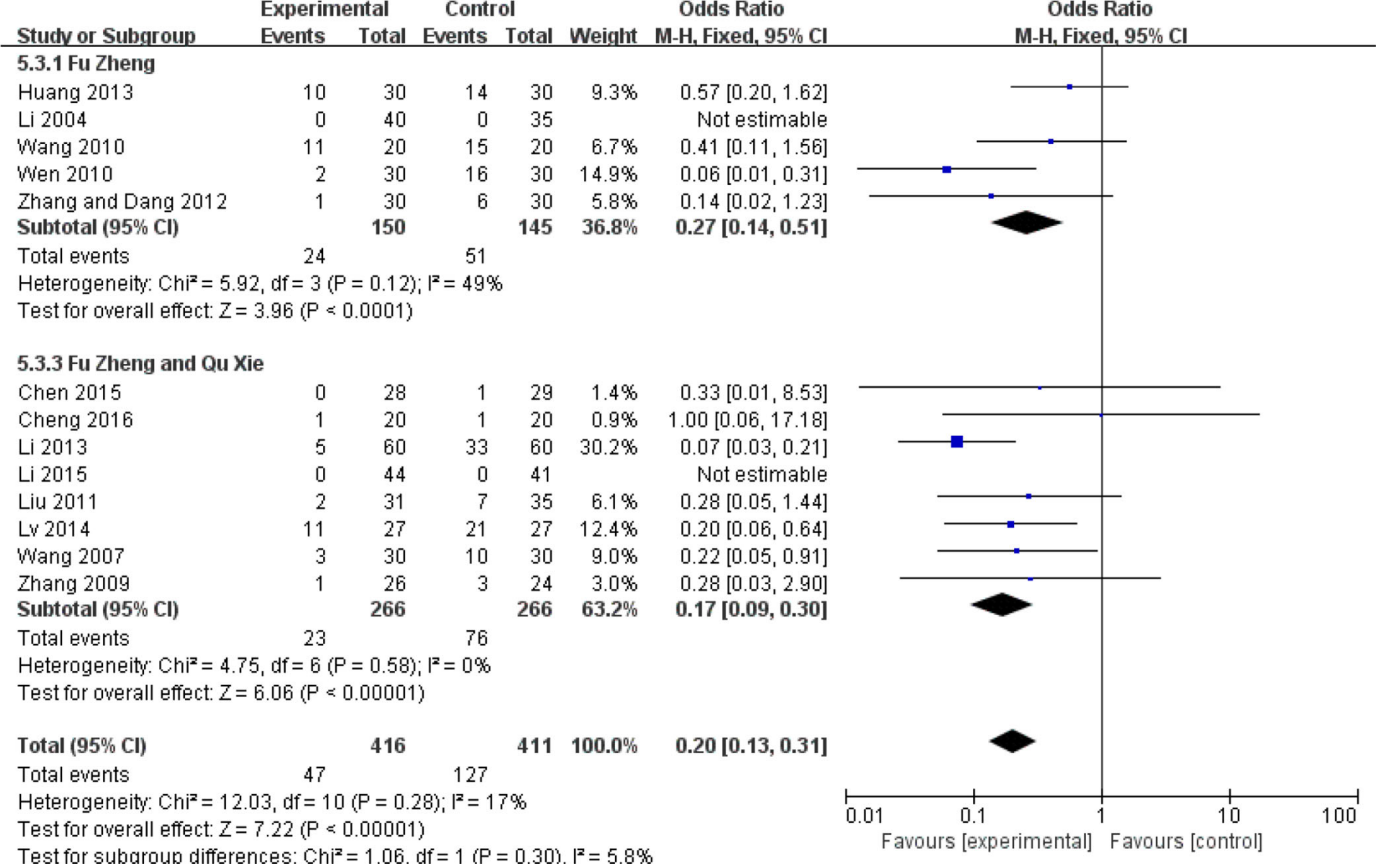
Forest plot of blood platelet reduction in patients of breast cancer (toxicity grades III–IV).

**Figure 17:**
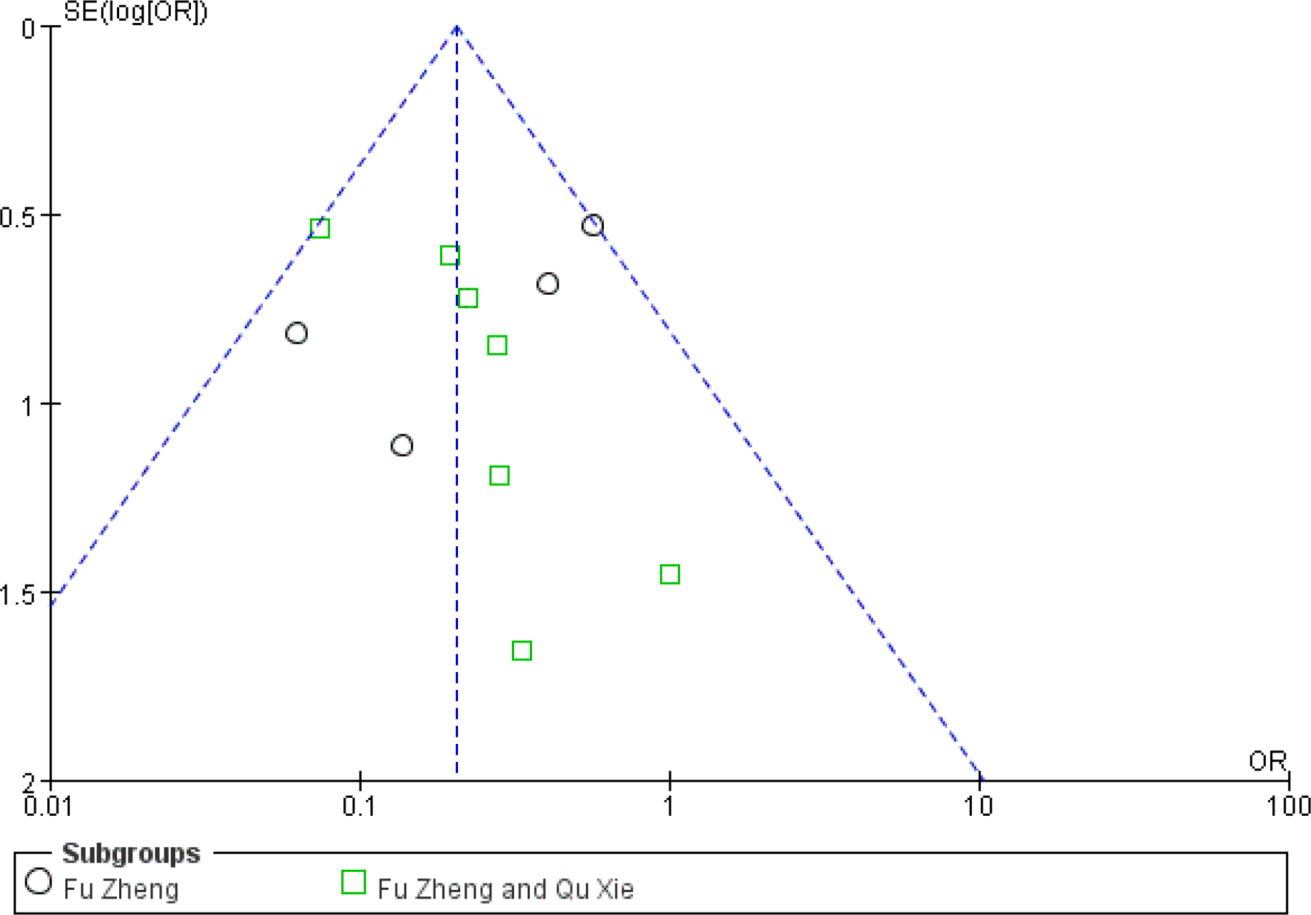
Funnel plot of blood platelet reduction in patients of breast cancer (toxicity grades III–IV).

The reduced white blood cell (WBC) and blood platelet count are used to assess myelosuppression, which is another common side-effect of chemotherapy, referring to impaired bone marrow activity (79, 80). For WBC reduction, the occurrence rate of grades III–IV was significantly reduced in the CHM groups (RR = 0.44, 95% CI = 0.35–0.55, I^2^ = 0%, 22 studies (19, 22, 24, 31, 32, 34–39, 42, 45, 46, 50–52, 57, 60, 61, 63, 69), 1,414 patients, **Figures 14** and **15**). Sub-group analysis showed that the occurrence rate of grades III–IV was significantly reduced in each sub-group. There is no statistical significance between these sub-groups (p = 0.18). Regarding blood platelet count, the reduction of blood platelet in the CHM groups is much less than that of the control groups (RR = 0.2, 95% CI=0.13–0.31, I^2^ = 17%, 11 studies (19, 22, 34, 35, 37, 39, 42, 45, 50–52, 61, 63), 827 patients, **Figures 16** and **17**).

### Impaired Immune Functions

**Figure 18:**
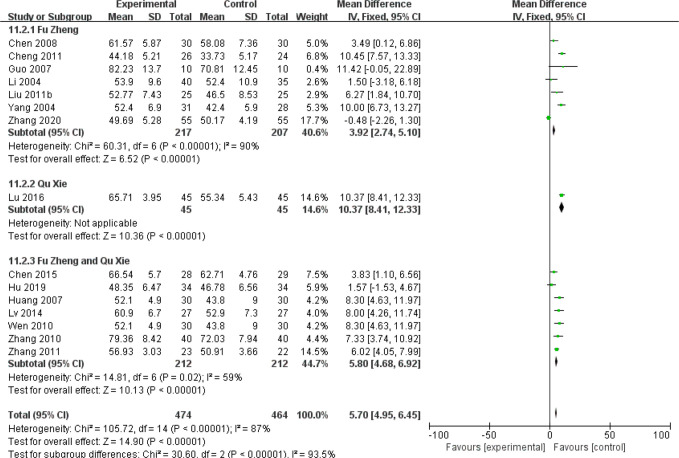
Forest plot of CD3+ in patients of breast cancer after chemotherapy.

**Figure 19:**
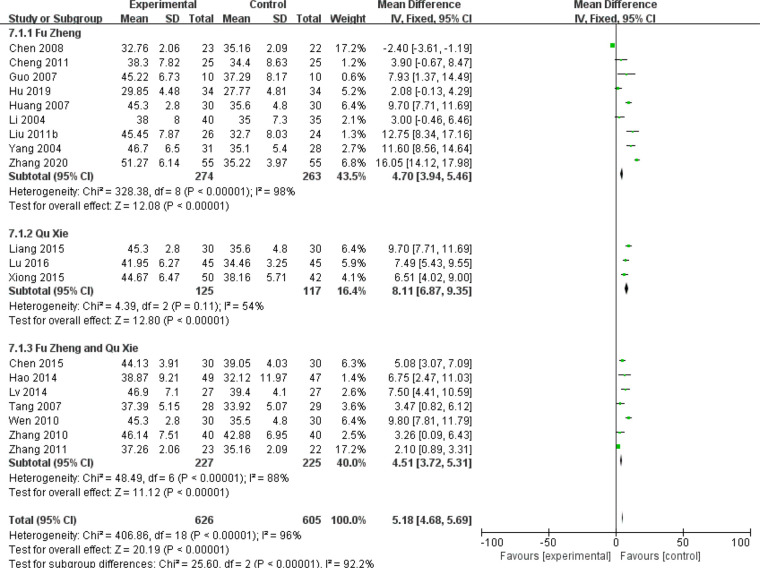
Forest plot of CD4+ in patients of breast cancer after chemotherapy.

**Figure 20:**
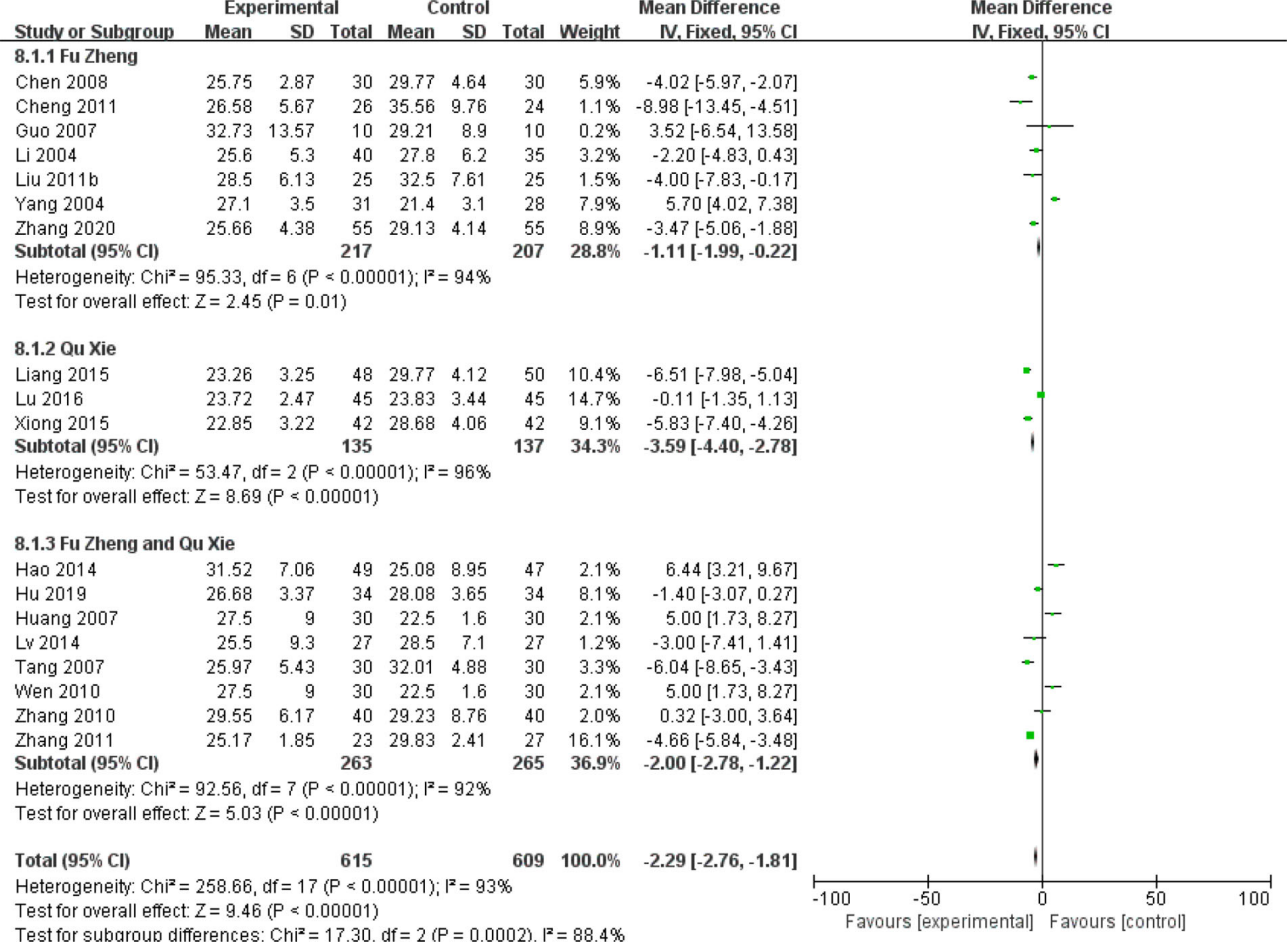
Forest plot of CD8+ in patients of breast cancer after chemotherapy.

**Figure 21:**
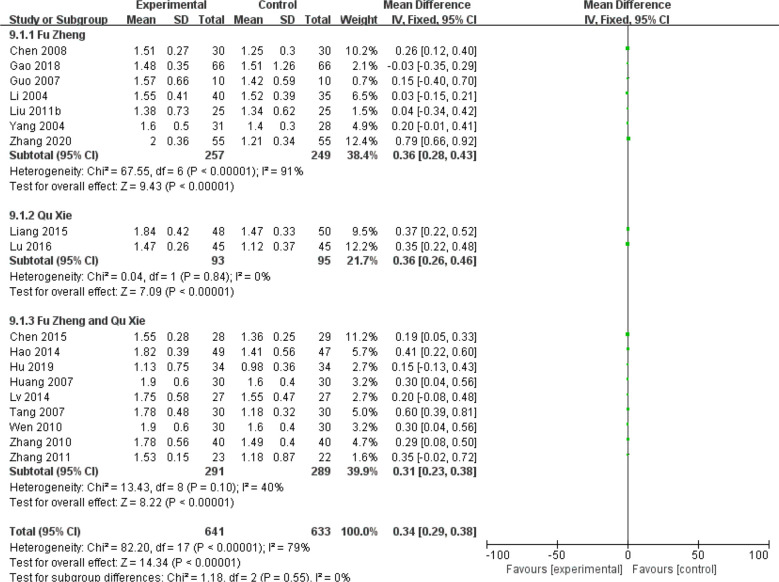
Forest plot of CD4+/CD8+ ratio in patients of breast cancer after chemotherapy.

**Figure 22:**
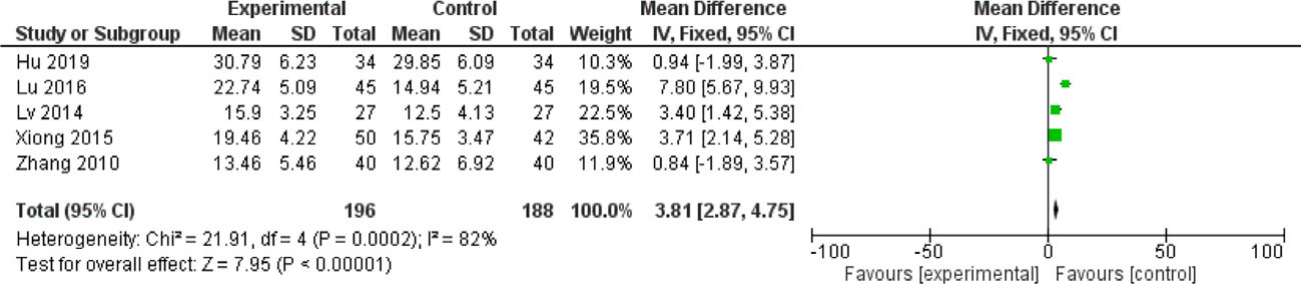
Forest plot of NK cells in patients of breast cancer after chemotherapy.

Chemotherapy would weaken the immune system (81). The impaired immune functions include the neutropenia, reduced T cells population and natural killer (NK) cells as well as serum IgG level (81, 82). Data of those cell population are continuous and mean difference analysis is adopted to decrease the influence of mean value difference. In terms of T cells, the population of CD3+ cells were significantly increased in patients with CHM intervention (MD = 5.7, 95% CI = 4.95–6.45, I^2^ = 87%, 15 studies (18, 19, 21, 26, 30, 33, 39, 43–45, 52, 55, 64–66), 938 patients, **Figure 18**). Regarding CD4+ cells (MD = 5.18, 95% CI = 4.68–5.69, I^2^ = 96%, 19 studies (18, 19, 21, 26, 28, 30, 33, 39, 41, 43–45, 52, 55, 64–66), 1,231 patients, **Figure 19**) and CD8+ cells (MD = −0.47, 95% CI = -0.59–0.34, I^2^ = 92%, 18 studies (18, 19, 21, 26, 28, 30, 33, 39, 41, 43–45, 52, 53, 55, 63, 65, 66), 1,124 patients, **Figure 20**) also increased in the CHM groups compared to that of the control group. Sub-group analysis showed that for each Fu Zheng, Qu Xie or Fu Zheng and Qu Xie sub-groups, the immune functions were significantly improved *via* CHM intervention. Interestingly, there is still high heterogeneity within each sub-group, which suggests there is another factor causing the heterogeneity of impaired immune functions. The ratio of CD4+/CD8+ also increased by CHM treatment (MD = 0.34, 95% CI = 0.29–0.38, I^2^ = 79%, 18 studies (18, 19, 25, 27, 28, 30, 33, 39, 41, 43–45, 49, 52, 55, 64–66), 1,274 patients, **Figure 21**). **Figure 22** indicated the population of NK cells are raised in breast cancer patients from the CHM groups (MD = 0.71, 95% CI = 0.50–0.92, I^2^ = 84%, 5 studies (30, 44, 45, 53, 65), 384 patients). However, of note, all of those studies showed high heterogeneity, which might result from the measure approaches, different cancer stages and age of patients, as well as sample size.

## Discussion

Currently, the treatment of breast cancer includes surgery, chemotherapy, radiation therapy, hormonal therapy, targeted therapy, and immunotherapy (3, 5), among which, chemotherapy is extensively used in not only early-stage invasive but also advanced-stage breast cancer, as well as before surgery to shrink the tumor in some cases (83). Generally, a combination of two or more chemotherapeutic agents is adopted as chemotherapy regimens for breast cancer (84). It shows remarkable efficacy in destroying cancer cells but also causes non-negligible adverse effects on patients, including nausea and vomiting, diarrhea, constipation, alopecia, myelosuppression, impaired immune function, *etc.* The severity of side-effects depends on the regimen type, dose used, treatment length, and general health of patients. Although several western medicines such as dexamethasone, 5-HT3 receptor antagonist, glutathione, magnesium sulfate and calcium gluconate have been used to reduce chemotherapy-induced side effects, they still hardly satisfy the requirements of patients (85). Thus, an adjuvant treatment to manage these side-effects is expected.

Increasing CHM is clinically used in breast cancer patients receiving chemotherapy for its beneficial effects in alleviating the side-effects. As a matter of fact, there are several systematic reviews and meta-analyses evaluating the efficacy of CHM combined with conventional therapy such as surgery, chemotherapy, or endocrine therapy for breast cancer (86–88). The tumor response, quality of life, hot flashes were particularly focused. In 2016, there are two review articles (86, 87) performed meta-analysis to assess CHM as adjunctive therapy to chemotherapy for breast cancer. Both of them did not get a confident conclusion due to the limited data. They did not identify as many studies as it could have. In this study, we have collected as many studies as we can and performed a meta-analysis to assess the efficacy of CHM in alleviating side-effects induced by chemotherapy in breast cancer patients. Plentiful studies have evaluated the tumor response and quality of life after adjunctive treatment of CHM and chemotherapy, but we focused on the adverse effects, and only studies with evaluation of chemotoxicity are included for meta-analysis in our study. All available data of those recruited studies were used without intentional selection. Regarding CINV, diarrhea, constipation, alopecia, and myelosuppression, dichotomy data is used to describe the frequency of adverse events. Although the criteria for classification are slightly different, those data from different studies showed low heterogeneity. Results showed that patients treated with different CHMs could significantly alleviate symptoms such as CINV, diarrhea, alopecia, and myelosuppression. In terms of the evaluation of immune function, the level of CD3+ T cells, CD4+ population, CD8+ population, CD4+/CD8+ ratio and the NK cell population are presented as continuous data. Although results after meta-analysis showed CHM intervention significantly improved T cells and NK cell population, significant heterogeneity was observed among these pooled studies. The different regimens, treatment duration, therapeutic dose, as well as age and cancer stage of patients might contribute to the high heterogeneity. To obtain more reliable conclusion, sub-group analysis according to chemotherapeutic regiments or cancer stage should be performed. In the present study, we performed sub-group analysis based on the principles of TCM treatment of breast cancer, which mainly include three aspects: strengthening the body (Fu Zheng, +), eliminating pathogenic factors (Qu Xie, −), or both (Fu Zheng and Qu Xie, ±). Sub-group analysis showed that for each Fu Zheng, Qu Xie or Fu Zheng and Qu Xie sub-groups, the immune functions were significantly improved *via* CHM intervention. Interestingly, there is still high heterogeneity within each sub-group, which suggests there is another factor causing the heterogeneity of impaired immune functions.

In this meta-analysis study, we have paid special attention to CINV, diarrhea, constipation, alopecia, myelosuppression, and reduced immune function, which are common symptoms of chemotherapy-associated adverse effect. Beside them, there are still many other symptoms which have been reported in breast cancer patients receiving chemotherapy, such as flash hot, kidney injury, liver injury, and cardiotoxicity from time to time (89, 90). However, the limited studies we obtained have clearly evaluated the efficacy of CHM on those adverse symptoms of chemotherapy-treated breast cancer patients due to the relatively low occurrence rate. In the study of Cheng and Zhang (22), they recruited 40 chemotherapy-treated breast cancer patients and assessed the relative level of aminotransferase in groups receiving Fuzheng Jiedu decoction or not. The results showed this decoction could slightly reduce the incidence rate of increased aminotransferase, two patients from the CHM group and four patients from the control group. In another study (59), the events of abnormal liver and kidney function in chemotherapy-treated breast cancer patients were recorded. There are two patients who reported abnormal liver and kidney function in the CHM group (Modified Fuzheng Xiaozheng Qudu decoction) while eight patients in the control group. Xu (54) has reported the effect of an herbal formula Sini Decoction on alleviating the anthracycline associated cardiotoxicity in breast cancer patients. This study claimed that Sini Decoction effectively slowed down cardiac toxicity and reduced the process of myocardial damage. More trials are needed to further confirm the positive role of CHM in ameliorating those chemotherapy-associated toxicities.

Though we strictly performed this meta-analysis according to review procedure claimed by the Cochrane Collaboration, this study has several limitations. First, the quality of pooled studies is generally poor. Most of these studies did not mention the allocation concealment and blinding of participants and personnel as well as blinding of outcome assessment, resulting in the unclear/high risk of selection bias, performance bias, and detection bias, which may overestimate the efficacy of intervention. Moreover, the detailed approaches of random grouping, follow-up and drop-out rate are not clear in most studies, particularly for Chinese papers. The details of sample size calculation are also missing in our pooled studies. Additionally, most of the studies haven’t provided placebo to patients of the control group, which may also lead to performance bias. Due to the nature of CHM, it is difficult to have placebo that could well mimic herbs (91). This is still a limitation and challenge of many trials of Chinese medicine. Secondly, there might exist publication bias in our enrolled studies. Almost all studies presented the positive data, but negative results might be unreported. Thirdly, the CHM intervention approaches are different from each other. In addition to the CHM composition (herbal formula, single herb, pure compound), the way to take the medicine (oral intake or intravenous injection) and dose used may contribute to high heterogeneity. Lastly, due to language barrier, only papers in English and Chinese are included in our study. Since CHM is also widely used in other Asian countries such as South Korea and Japan, additional studies published in their local language should also be considered.

At present, although the usage of CHM in the treatment of breast cancer has been described for thousands of years in China, accepting CHM remains a challenge nowadays in western countries. One of the reasons is due to language barrier. Most clinical trials regarding the use of CHM on breast cancer are reported in Chinese. Thus, more clinical trials reported in English are warranted to facilitate the globalization of Chinese Medicine. Another concern raised from scientists and physicians is whether CHM would have an influence on the pharmacokinetics or anti-cancer effect of chemotherapeutic regimens when used in combination (92). In some animal studies, it is reported that some of CHM such as berberine showed no interaction with chemotherapeutic agents and did not reduce the anti-tumor efficacy (93–95). In clinic trials, however, few studies performed pharmacokinetics evaluation of chemotherapeutic agents in patients receiving CHM treatment. Last but not least, scientific evidence of molecular mechanism underlying its beneficial effects on chemotherapy induced adverse effects is required to comprehensively understand and more appropriately use CHM in clinical for breast cancer.

## Conclusion

Because of the limitations of low quality of the enrolled trials, it is hard to reach a firm conclusion. Nevertheless, our work suggested the potencies of CHM to facilitate the management of chemotherapy toxicity, and more efforts are needed to promote the application of CHM in the clinic. Larger sample size, double-blinded, randomized, placebo-controlled clinical trials with modern and rigorous methodology are expected to offer more convincing evidence to show the efficacy and safety of CHM in the management of chemotherapy-associated side-effects. Moreover, it is of great importance to further observe whether CHM has an influence on the metabolism of chemotherapeutic drugs and anti-tumor effects in clinical trial. In addition, more efforts should be made in identifying molecular signaling pathways involved in chemotoxicity in breast cancer patients, which could facilitate the understanding of the role of CHM in the management of cancer treatment.

## Data Availability Statement

The original contributions presented in the study are included in the article/supplementary material. Further inquiries can be directed to the corresponding authors.

## Author Contributions

YF and EY conceived and designed the study. SL and T-hS developed the search terms and drafted the manuscript. GT, H-YT, and NW reviewed the protocol and revised the manuscript. BN and CC initiated the idea and revised the manuscript. All authors contributed to the article and approved the submitted version.

## Funding

The study was financially supported by Innovation and Technology Commission: The 2^nd^ Phase of Integrative Joint Organizational Platform (IJOP) Disease Collaborative Panel (Project Code: 200009062), Wong’s Donation (Project Code: 200006276), the Gaia Family Trust for Modern Oncology of Chinese Medicine (Project Code: 200007008), Research Grant Council, HKSAR (Project Code RGC GRF 17152116), and Health and Medical Research Fund (Project Codes 15162961, 16172751 and 17181101).

## Conflict of Interest

The authors declare that the research was conducted in the absence of any commercial or financial relationships that could be construed as a potential conflict of interest.
